# New transitional fossil from late Jurassic of Chile sheds light on the origin of modern crocodiles

**DOI:** 10.1038/s41598-021-93994-z

**Published:** 2021-07-22

**Authors:** Fernando E. Novas, Federico L. Agnolin, Gabriel L. Lio, Sebastián Rozadilla, Manuel Suárez, Rita de la Cruz, Ismar de Souza Carvalho, David Rubilar-Rogers, Marcelo P. Isasi

**Affiliations:** 1 Laboratorio de Anatomía Comparada y Evolución de los Vertebrados (LACEV) Museo Argentino de Ciencias Naturales “Bernardino Rivadavia” (MACN), Av. Ángel Gallardo 470 (C1405DJR), Buenos Aires, Argentina; 2 Consejo Nacional de Investigaciones Científicas y Técnicas (CONICET), Buenos Aires, Argentina; 3 Fundación de Historia Natural “Félix de Azara”, Centro de Ciencias Naturales, Ambientales y Antropológicas, Universidad Maimónides, Hidalgo 775 (C1405BDB), Buenos Aires, Argentina; 4 Universidad Andres Bello, Carrera de Geología, Avenida República 237, Santiago, Chile; 5 Servicio Nacional de Geología y Minería, Avenida Santa María 0104, Santiago, Chile; 6 Universidade Federal do Rio de Janeiro, Departamento de Geologia, CCMN/GEO, Cidade Universitária, Ilha do Fundão, Rio de Janeiro, RJ 21.910-200 Brazil; 7 Área Paleontología, Museo Nacional de Historia Natural, Casilla 787, Santiago, Chile; 8 Universidade de Coimbra, Centro de Geociências, Rua Sílvio Lima, 3030-790 Coimbra, Portugal

**Keywords:** Evolution, Zoology, Palaeontology

## Abstract

We describe the basal mesoeucrocodylian *Burkesuchus mallingrandensis* nov. gen. et sp.*,* from the Upper Jurassic (Tithonian) Toqui Formation of southern Chile. The new taxon constitutes one of the few records of non-pelagic Jurassic crocodyliforms for the entire South American continent. *Burkesuchus* was found on the same levels that yielded titanosauriform and diplodocoid sauropods and the herbivore theropod *Chilesaurus diegosuarezi*, thus expanding the taxonomic composition of currently poorly known Jurassic reptilian faunas from Patagonia. *Burkesuchus* was a small-sized crocodyliform (estimated length 70 cm), with a cranium that is dorsoventrally depressed and transversely wide posteriorly and distinguished by a posteroventrally flexed wing-like squamosal. A well-defined longitudinal groove runs along the lateral edge of the postorbital and squamosal, indicative of a anteroposteriorly extensive upper earlid. Phylogenetic analysis supports *Burkesuchus* as a basal member of Mesoeucrocodylia. This new discovery expands the meagre record of non-pelagic representatives of this clade for the Jurassic Period, and together with *Batrachomimus*, from Upper Jurassic beds of Brazil, supports the idea that South America represented a cradle for the evolution of derived crocodyliforms during the Late Jurassic.

## Introduction

In contrast to the Cretaceous Period and Cenozoic Era, crocodyliforms from the Jurassic Period are predominantly known from marine forms (e.g., thalattosuchians)^1^. Much less is known about non-pelagic crocodyliforms from this time span, complicating our understanding of diversification patterns and the origin of eusuchians and the line to modern crocodylians^2–4^.

Jurassic non-pelagic crocodyliforms are represented by protosuchian-grade taxa and, on the other hand, by modern aspect neosuchians such as Goniopholididae, Atoposauridae and Pholidosauridae^1,5–9^. In the case of South America, Jurassic non-thalattosuchian crocodyliforms are solely represented by the putative paralligatorid *Batrachomimus* from the Upper Jurassic of Brazil^10^. The paucity of the Jurassic crocodyliform fossil record of non-pelagic forms is one of the main reasons for uncertainties on the morphological changes that occurred between basal “protosuchians” and more derived mesoeucrocodylians.

The aim of the present contribution is to provide a preliminary description of *Burkesuchus*, emphasizing the particular combination of both derived and plesiomorphic characters. The new taxon helps fill the morphological gap between “protosuchian” grade and early branching mesoeucrocodylian crocodyliforms.

## Systematic paleontology

Archosauria Cope, 1869.

Crocodyliformes Hay, 1930.

Mesoeucrocodylia Whetstone and Whybrow, 1980.

*Burkesuchus mallingrandensis* nov. gen. et sp.

zoobank.org:pub:0F91A36B-1379-41CE-AB54-7B94542D8539.

### Holotype

**Figure 1:**
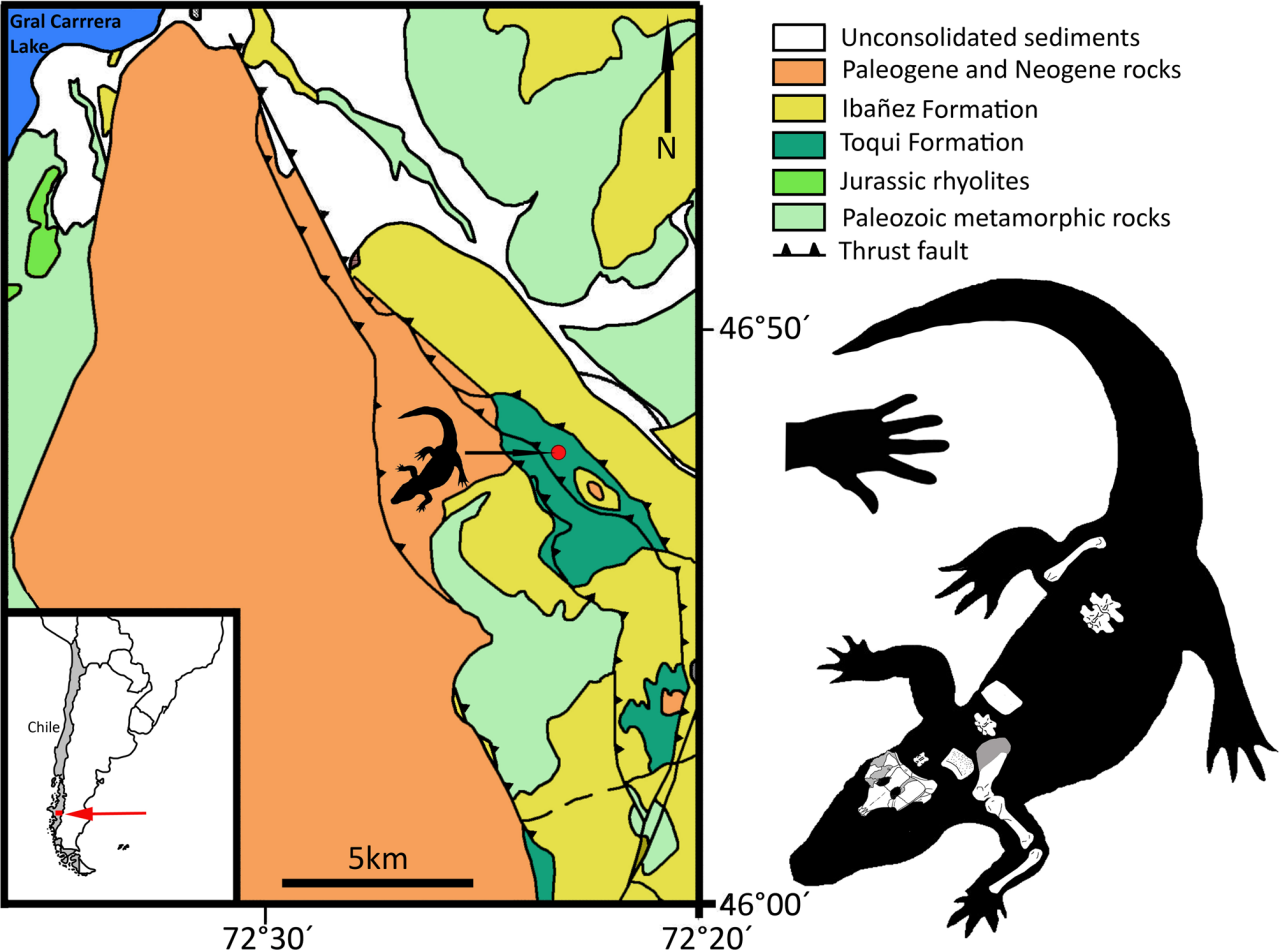
Locality map, geological context, and skeletal reconstruction of *Burkesuchus mallingrandensis*. Skeletal reconstruction based on holotype and paratype specimens.

SGO.PV 17700 (Colección Paleontología Vertebrados, Museo Nacional de Historia Natural, Santiago, Chile), partial skeleton including partial neurocranium, a cervical neural arch, four dorsal vertebrae, right scapula and coracoid, right humerus and ulna, left ischium, distal end of right femur, and one cervical and two dorsal osteoderms (Fig. 1; Table 1; Supplementary Information).

**Table 1:** Measurements (in mm) of selected elements of the holotype specimen of *Burkesuchus inialen* nov. gen. et sp. (SGO.PV 17700).

**Cranium**	
Distance from anterior tip of frontals up to supraoccipital	556
Maximum transverse width at level of squamosals (reconstructed)	680
Maximum transverse width at level of quadrates (reconstructed)	740
Quadrate length:	400
**Scapular girdle and forelimb**	
Anteroposterior diameter of proximal end of scapula	140
Proximodistal height of coracoid	280
Anteroposterior length of distal end of coracoid	140
Humerus length	520
Humerus proximal transverse width	200
Humerus distal transverse width	130
Ulna length	530
**Osteoderm**	
Cervical osteoderm transverse width	220
Cervical osteoderm anteroposterior length	120

### Paratype

SGO.PV 17701, nearly complete right femur, two dorsal vertebrae, and one dorsal osteoderm (Fig. 1; Table 1).

### Diagnosis

Small-sized crocodyliform diagnosed on the following combination of characters (autapomorphies marked by an asterisk*): cranial roof bones ornamented by grooves and pits; frontals fused and subtriangular in contour, with strongly convergent lateral margins anteriorly; frontals anteroposteriorly short (transverse width representing approximately 90% of its length)*; squamosal posteroventrally flexed forming a wide bony wing and delimiting the posterior opening of the meatal chamber, which is reduced to a small duct*; supratemporal foramen small; squamosal and quadrate widely exposed on occipital surface of cranium; paraoccipital processes of otoccipital relatively small; foramina for cranial nerves IX-XII dorsally limited by the paraoccipital process; and dorsal vertebrae with kidney-shaped prezygapophyses.

### Etymology

Genus name honours Mr. Coleman Burke (New York, USA), who generously supported the field exploration in which the fossils were discovered; and *suchus*, from Latin, crocodile; species name *mallingrandensis*, refers to Mallín Grande, a beautiful region in southern Chile adjacent to the fossil locality.

### Locality and horizon

The holotype and referred specimens of *Burkesuchus* were collected from beds of the Toqui Formation, cropping out in the mountains flanked by the Maitenes and Horquetas rivers, south of General Carrera Lake (Fig. 1). The rock succession consists of a 300–320 m thick sequence of conglomerates with intercalated tuffs. *Burkesuchus* fossils occur in an approximately 100 m succession of alternating green volcaniclastic pebbly sandstones and sandy sedimentary breccias, with intercalations of lapilli tuffs and red ignimbrites with eroded tops. The U-Pb SHRIMP age of 147 ± 1.0 Ma was obtained from zircon samples from the ignimbrite that immediately underlies the fossil-bearing levels, indicating a Tithonian age (latest Jurassic) for *Burkesuchus* and its associated fauna^11,12^. Other fossil vertebrates currently documented from these beds include titanosauriform and diplodocoid sauropods, along with the herbivorous theropod *Chilesaurus diegosuarezi*^13–15^.

### Description

Available specimens of *Burkesuchus* indicate a relatively small animal roughly 70 cm long; this is based on comparisons with complete skeletons of *Protosuchus richardosoni*^16^ (see Table 1).

### Cranium

**Figure 2:**
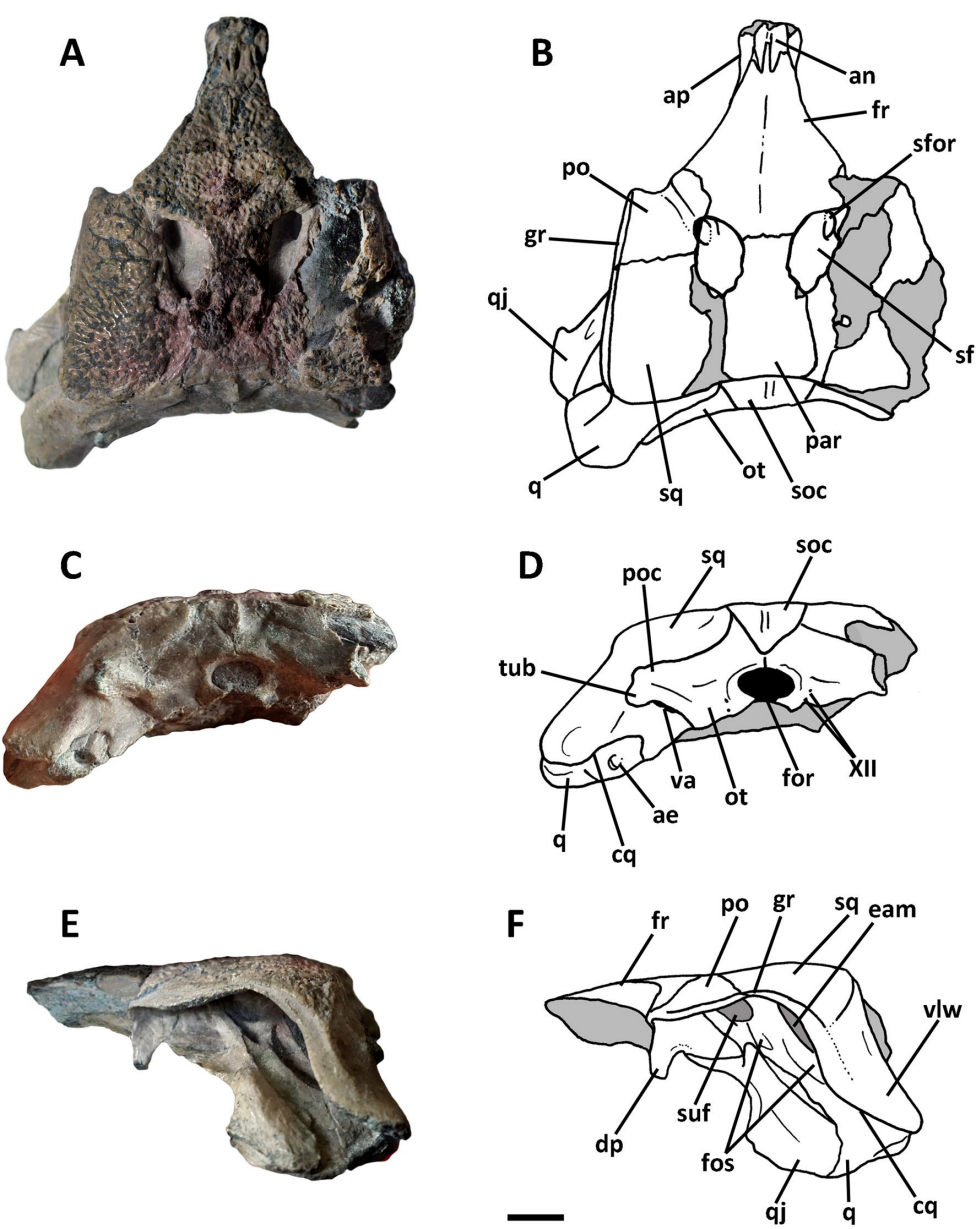
Photographs and line drawings of the cranium (SGO.PV 17700) of *Burkesuchus mallingrandensis* in (**A**, **B**) dorsal; (**C**,**D**) posterior; and (**E**,**F**) left lateral views. ae, foramen aereum; an, surface for articulation with nasal; ap, surface for articulation with prefrontal; cq, cranioquadrate passage; dp, descending process of the postorbital; eam, external auditory meatus; for, foramen magnum; fos, blind fossae; fr, frontal; gr, longitudinal groove for the upper earlid; ot, otoccipital; par, parietal; q, quadrate; qj, quadratojugal; par, parietal; po, postorbital; poc, paraoccipital process; sf, supratemporal fenestra; sfor, supratemporal foramen; soc, supraoccipital; sq, squamosal; suf, subtympanic foramen; tub, ventral tubercle of paraoccipital process; va, vagi foramen; vlw, ventrolateral wing of the squamosal; XII, exit foramina for the hypoglossal nerve. Scale bar: 1 cm.

*Burkesuchus* exhibits a dorsoventrally compressed braincase and cranium that is transversely wide at the posterior margin. The posterior margin of the squamosal, quadrate and quadratojugal slope strongly posteroventrally, a condition different from the subvertical orientation present in “protosuchians” and notosuchians^17,18^ (Fig. 2E,F). The cranial roof is strongly ornamented by pits and grooves (Fig. 2A,B). The frontals are fused and show a midline longitudinal ridge, as in notosuchians and basal neosuchians^19^. In dorsal view the frontals are subtriangular in contour with strongly anteriorly convergent lateral margins. The contact with the nasals is interdigitated, forming a “W”-shaped suture. The frontals are notably short and delimit the anteromedial margin of the supratemporal fenestrae. The fenestrae are proportionally small and ovoid in contour, similar to *Sichuanosuchus*^20^. The supratemporal fossae of *Burkesuchus* exhibit a small supratemporal foramen at the anterolateral margin. The laterosphenoid is widely exposed in the supratemporal fossa. In dorsal view the postorbital is gently convex and is anteroposteriorly short, representing half the length of the squamosal. It shows a squared-off anterolateral margin with a short projection, and a concave anterior margin. In dorsal view, the cranial table is transversely wide.

In lateral view the postorbital shows a rod-like descending process, as typical in modern crocodilians^4,19,21^. The postorbital contact for the quadratojugal is narrow, constituting a derived mesoeucrocodylian condition^22,23^. The quadratojugal looks stout and anteroposteriorly expanded. The anterodorsal corner of the quadratojugal exhibits a well-defined excavation, which probably hosted the fleshy component of *m. depressor auricular superior*, *m. levator auricular superior*, and the lining muscle responsible for movements of the upper earlids^24,25^. The quadrate is notably elongate and posteroventrally extended, and shows a pair of deep excavations along its dorsal surface (albeit not fully laterally facing as in “protosuchians”^21^). The subtympanic foramen is wide, well-defined and located anterodorsally with respect to the otic incisure. A narrow ridge along the dorsal margin of the quadrate is here considered as the possible ventral limit of the periotic fossa, and thus, as the anterior extension of the tympanic membrane.

A well-defined longitudinal groove runs along the lateral edge of postorbital and squamosal, indicative of an anteroposteriorly extensive upper earlid^21^. The squamosal is strongly flexed posteroventrally, forming an expanded wing that partially covers the meatal chamber. The squamosal wing delimits the posterior opening of the meatal chamber, which is reduced to a small duct. The external auditory meatus is deeply sunk into the squamosal, being mostly covered laterally by this bone.

On the posteromedial corner of quadrate there exist a conspicuous *foramen aereum*. The squamosal and quadrate are in near contact with one another along their posterior surfaces, leaving a small canal that may represent a precursor of the eusuchian cranioquadrate foramen^26^.

The occipital surface faces posterodorsally. The supraoccipital is small, subtriangular in contour and with a prominent nuchal crest. The otoccipital is relatively small, and lacks the expanded ventrolateral surface present in “protosuchians”^22^. The paraoccipital processes are dorsoventrally narrow and bear a small lateroventral tubercle, as in some basal neosuchian crocodyliforms^3,27^. The squamosal is widely exposed on the occipital plane (Fig. 2C,D), extending laterally and ventrally well beyond the level of paraoccipital processes. This peculiar condition of the squamosal appears unique in *Burkesuchus* among crocodyliforms. An expanded squamosal may be also observed in the “protosuchians” *Orthosuchus* and “*Notochampsa*”, but it is not sigmoid in contour, and is not strongly deflected nor appressed to the quadrate^23^. The paraoccipital process delimits the vagi foramina (including the openings of the IX, X, XI nerves). Cranial nerve XII exhibits a double exit. The foramen magnum is transversely wide and is dorsally delimited by crests.

### Vertebrae

**Figure 3:**
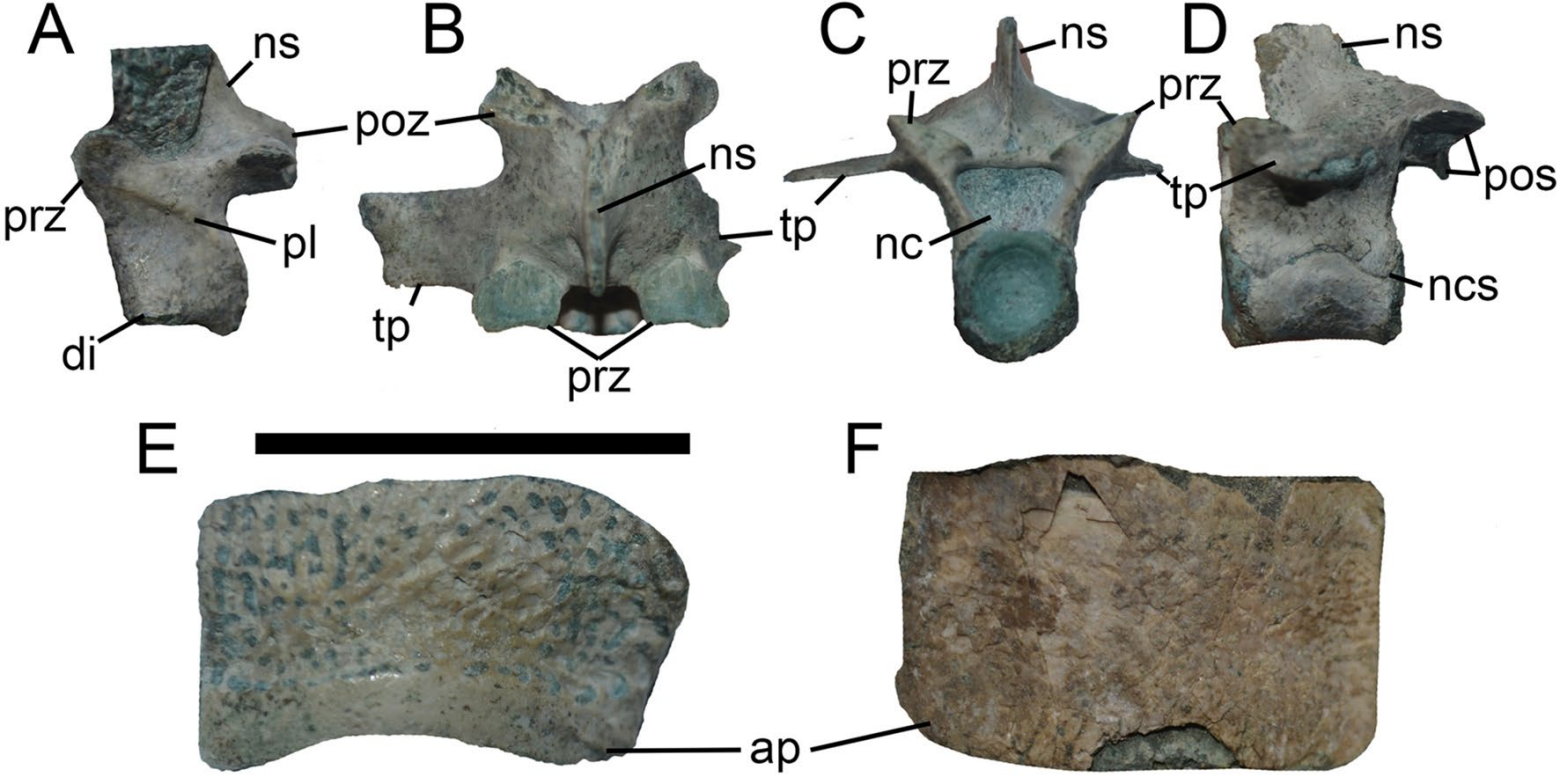
Photographs of vertebrae and osteoderm of *Burkesuchus mallingrandensis* (SGO.PV 17700). (**A**) cervical vertebra in left lateral view; (**B**–**D**) dorsal vertebra in (**B**) dorsal, (**C**) anterior, and (**D**) left lateral views; (**E**–**F**), dorsal osteoderm in (**E**), dorsal and (**F**), ventral views. ap, articular facet for the preceding osteoderm; di, diapophysis; nc, neural canal; ns, neural spine; pl, centroprezygapophyseal lamina; poz, postzygapophysis; prz, prezygapophysis; tp, transverse process. Scale bar: 1 cm.

The available cervical neural arch is dorsoventrally tall and with a high neural spine that is anteriorly inclined. The postspinal fossa is well-excavated and teardrop-shaped. In lateral view, a well-developed accessory centroprezygapophyseal lamina is present (Fig. 3A). Dorsal vertebrae are amphicoelous, with articular surfaces of centra sub-circular in outline, as occurs in neosuchians^4^. Neural arches are dorsoventrally tall, and have a long, sub-rectangular shaped neural spine, representing more than twice the dorsoventral height of respective centrum (Fig. 3B–D). The neural canal is notably wide. There are no spinoprezygapophyseal or spinopostzygapophyseal laminae. The articular surfaces of the prezygapophyses are transversely wider than anteroposteriorly long and reniform in contour, with a notched anterior margin. A well-developed interpostzygapophyseal lamina is present.

### Pectoral girdle

**Figure 4:**
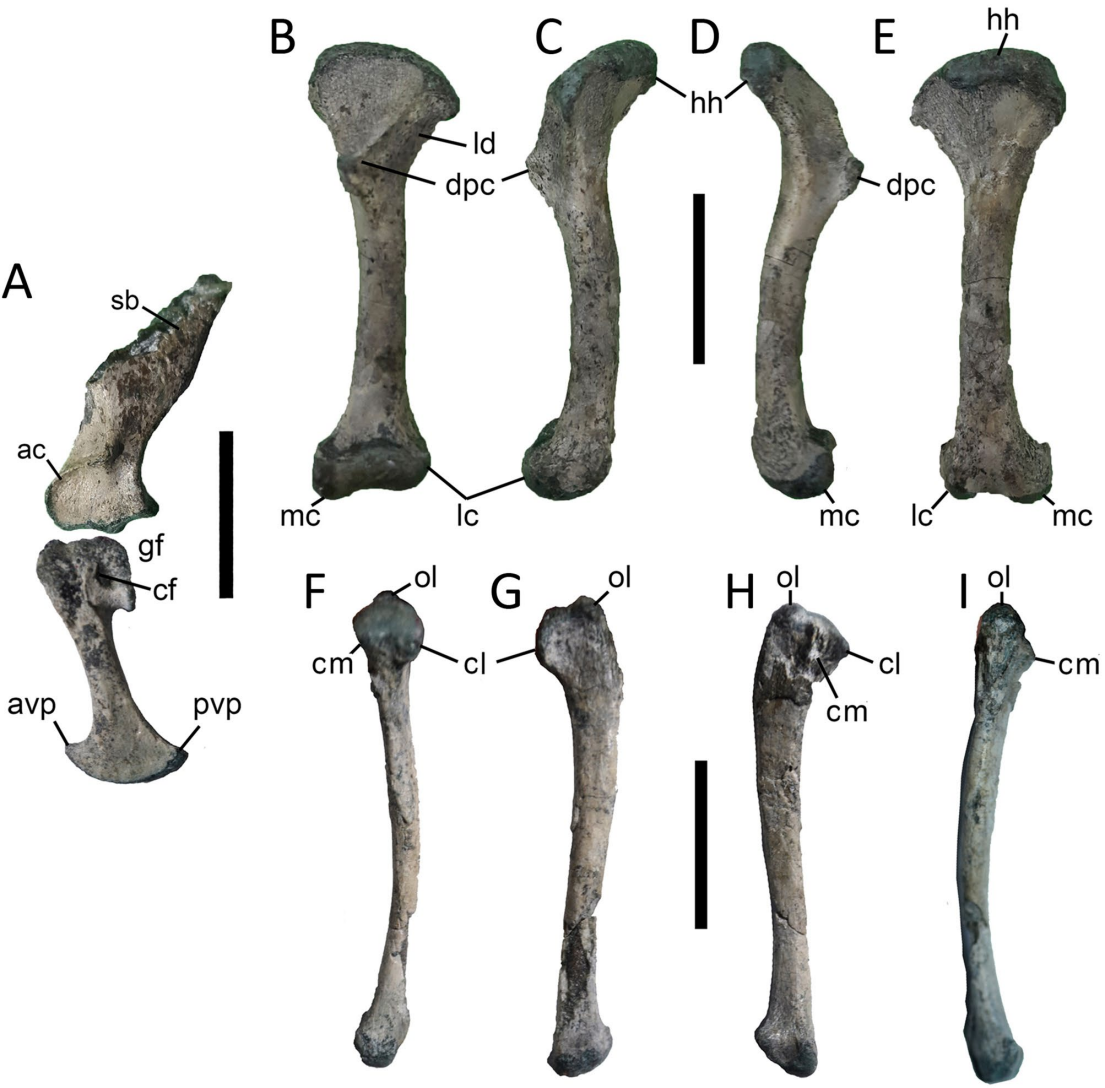
Photographs of pectoral girdle and forelimb bones of *Burkesuchus mallingrandensis* (SGO.PV 17700). (**A**) Left scapula and coracoid in lateral view; (**B**–**E**), left humerus in (**B**) anterior, (**C**) lateral, (**D**) medial, and (**E**) posterior views; (**F**–**I**) left ulna in (**F**) anterior, (**G**) lateral, (**H**) medial, and (**I**) posterior views. ac, acromion; avp, anteroventral process; cf, coracoid foramen; cl, lateral cotyle; cm, medial cotyle; dpc, deltopectoral crest; hh, humeral head; gf, glenoid facets; lc, lateral condyle; ld, lateral depression; mc, medial condyle; ol, olecranon; pvp, posteroventral process; sb, scapular blade. Scale bar: 2 cm.

The scapula is transversely robust (Fig. 4A). As in basal crocodyliforms (e.g., *Orthosuchus*^23^), the acromial process of *Burkesuchus* is prominent and associated with a well-developed acromial ridge that is distally extended through the centre of the scapular blade. The glenoid facet is ventrally facing and dorsally bound by a prominent lip, a condition reminiscent of certain mesoeucrocodylians (e.g., *Notosuchus, Yacarerani, Caiman*^28^). In contrast, the coracoidal portion of the glenoid facet is posteriorly oriented and devoid of prominent edges. This later condition resembles that of basal crocodyliforms (e.g., *Orthosuchus*^23^) and differs from the posterodorsally oriented coracoidal glenoid facet of mesoeucrocodylians (e.g., *Notosuchus, Yacarerani, Caiman*^28^). The ventral process of coracoid is notably elongate and relatively narrow, with a strongly expanded distal end.

### Forelimb

The length of humerus represents 68% of the maximum width of the cranium, compared with 75% in *Protosuchus*^16^. It is straight in cranial view, but gently sigmoid in side view (Fig. 4B–E). The humerus of *Burkesuchus* shows a prominent proximolateral expansion, as occurs in mesoeucrocodylians^29^. However, its proximal end lacks features diagnostic of notosuchians (e.g., presence of a deep circular depression on the posterior surface of the proximal humerus, medially displaced proximal one-third of the deltopectoral crest, with a medially tilted distal end, and deltopectoral crest anterolaterally delimited by a well-defined concavity)^28^. The deltopectoral crest is prominent, subtriangular in lateral view and strongly anteromedially projected, as occurs in *Orthosuchus*^23^. The ulna is transversely compressed and shows a proximally rounded and well-defined olecranon (Fig. 4F–I). The proximal end of ulna exhibits well-developed proximal cotyles and an intercotylar crest and process, suggesting the presence of complex elbow movements, as in extant crocodilians^30^. It lacks the processes to accommodate the radius, thus differing from ziphosuchians^28^.

### Hind limb

**Figure 5:**
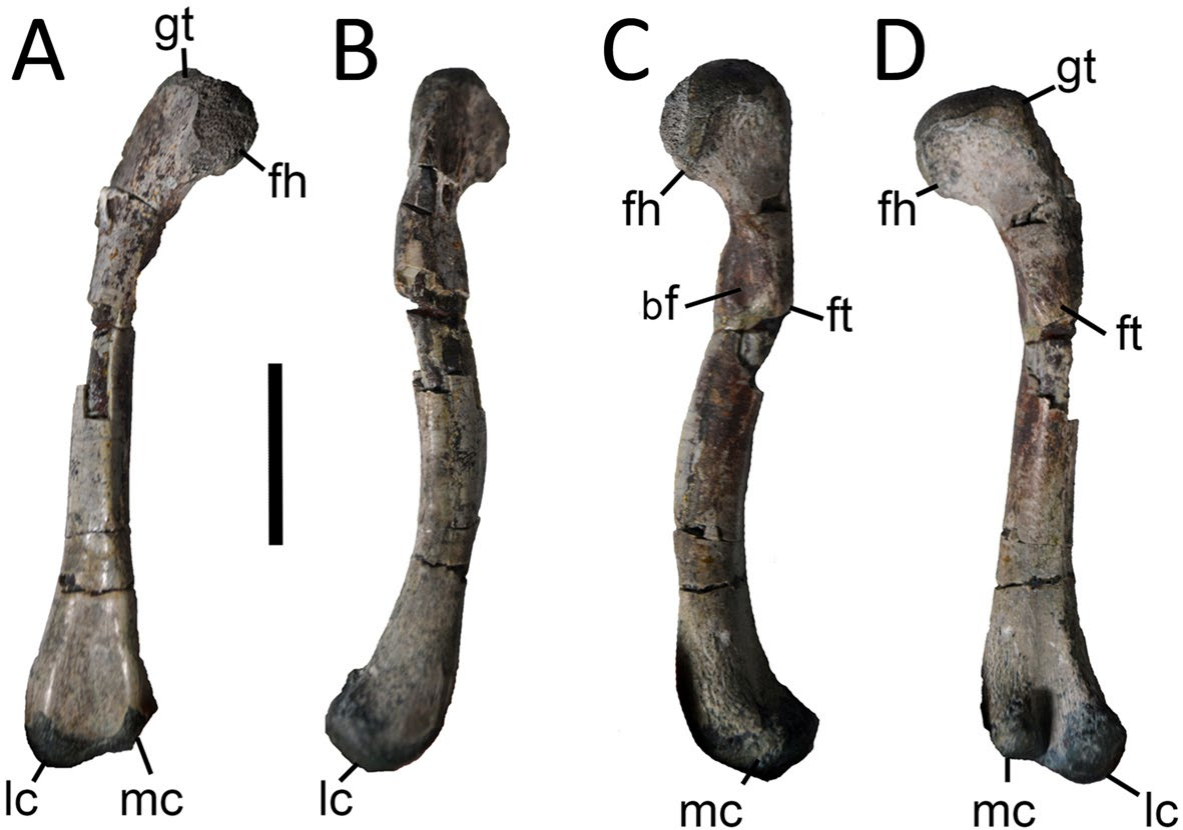
Photographs of right femur of *Burkesuchus mallingrandensis* (SGO.PV 17701) in (**A**) anterior, (**B**) lateral, (**C**) medial, and (**D**) posterior views. bf, basitrochanteric fossa; ft, fourth trochanter; gt, greater trochanter; fh, femoral head; lc, lateral condyle; mc, medial condyle. Scale bar: 2 cm.

The femur is known from a referred specimen (Fig. 5). Its shaft is relatively gracile and sigmoid in all views, with major axes of both proximal and distal ends meeting at an angle close to 50°. This condition is intermediate between that of non-eusuchian crocodyliforms (between 17° and 45°^31^) and living crocodiles (between 60° and 65°^32^). The 4th trochanter is prominent and the basitrochanteric fossa is deep and well-defined, indicating a deep anchoring for the *Mm. caudofemoralis longus* and *brevis*. The distal end of the bone exhibits highly asymmetrical distal condyles that are posterolaterally oriented. The lateral condyle is about two times larger than the medial condyle and is more ventrally extended, resulting in an asymmetric distal femur.

### Osteoderms

Cervical osteoderms are subquadrangular in contour, with the lateral third ventrally inclined (Fig. 3E,F). Trunk osteoderms, instead, are subrectangular in contour, with the anteroposterior length being half the transverse width. They are devoid of a dorsal keel and anterolateral prongs. Cranially, they exhibit a well-defined articular facet for the preceding osteoderm, representing approximately 1/3 of its entire length. The dorsal surface is ornamented by small, randomly distributed pits. Notably, trunk osteoderms are considerably enlarged with respect to the remaining skeletal elements. For example, they are transversely as wide as the cranium, a condition different from basal crocodyliforms (e.g., *Protosuchus*^16^) in which the osteoderms represent a quarter of the posterior transverse width of cranium. In this regard, *Burkesuchus* is closer to the proportions seen in basal mesoeucrocodylians (e.g., *Sarcosuchus*^33^).

## Discussion

### Phylogenetic position of *Burkesuchus*

**Figure 6:**
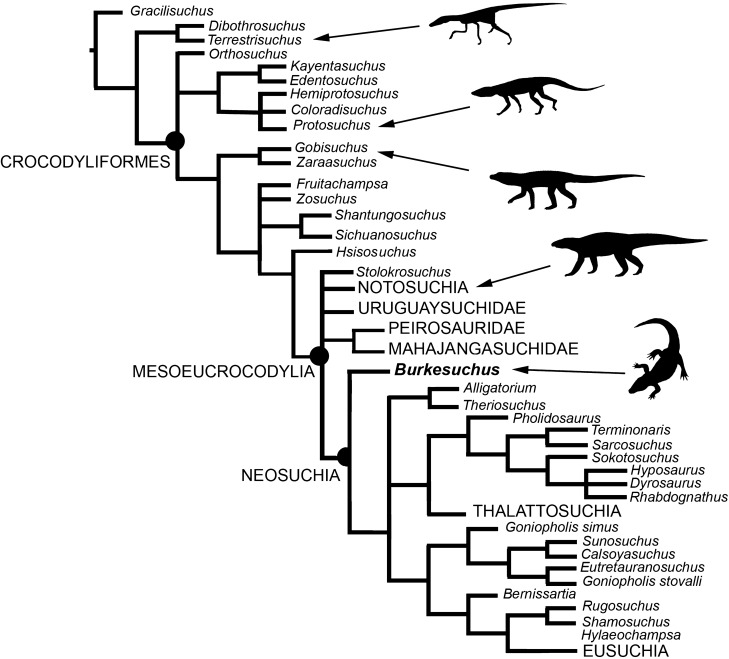
Cladogram showing the phylogenetic position of *Burkesuchus mallingrandensis*. See details in Supplementary Information.

The new taxon was included in a comprehensive phylogenetic analysis including most crocodyliforms^34^ (see Supplementary Online Material and Supplementary Online Material-Data Matrix; Fig. 6). The analysis resulted in the nesting of *Burkesuchus* among mesoeucrocodylians, exhibiting the following synapomorphies of this clade: squamosal with laterally oriented groove for the upper earlid, quadratojugal with narrow contact with the postorbital, postorbital delimiting the infratemporal fenestra, single fenestra (external auditory meatus) on the quadrate, quadrate lacking of a longitudinal keel, and supraoccipital relatively small and lacking dorsal exposure^3,4,19,21,35^ (see details on Supplementary Information). In contrast to both “protosuchians” and notosuchians, *Burkesuchus* exhibits a postorbital with a rod-like descending process, dorsoventrally low meatal chamber and reduced quadrate pneumaticity, and dorsal end of quadrate strongly forwardly oriented^18,36^, constituting synapomorphies uniting the Chilean taxon with Eusuchia. However, *Burkesuchus* retains several plesiomorphic features reminiscent of “protosuchians”, which support its position outside Eusuchia, including: relatively small and subtriangular-shaped frontals that contribute little to the margin of the supratemporal fenestra; reduced postorbital when compared with squamosal in dorsal view; and supratemporal foramen relatively small and located at the anterolateral corner of the supratemporal fenestra^36,37^.

### *Burkesuchus* and its implications for the evolution of meatal chamber in mesoeucrocodylians

Skull anatomy of *Burkesuchus* looks intermediate between that of “protosuchians” and neosuchian crocodyliforms with regard to the meatal chamber, earlid position, and general cranial shape.

*Burkesuchus* is similar to derived mesoeucrocodylians in exhibiting a meatal chamber that is anteriorly dorsoventrally low (due to the reduction of both the anterodorsal process of quadratojugal and descending process of postorbital), the posterior closure of the meatal chamber (due to of the posteroventral flexing of the squamosal and its close relation with the quadrate), and in having an extensive groove for the earlid on both the postorbital and squamosal. This morphology sharply contrasts with that of “protosuchians” (e.g., *Protosuchus, Hemiprotosuchus, Orthosuchus*) in which the meatal chamber is dorsoventrally deep, the external auditory meatus is completely opened posteriorly, and the sulcus for the upper earlid is not posteriorly extended on the lateral margin of squamosal^21,36,37^. *Burkesuchus* also differs from basal notosuchians (e.g., *Araripesuchus*), in which the meatal chamber is expanded and strongly concave^21^. It also shares some similarities with the baurusuchid pattern, as represented by *Pissarrachampsa*^21^, including: deflected posteroventral prong of the squamosal, posterior extension of the sulcus for the upper earlid along this posteroventral prong, presence of several pneumatic apertures on the anterodorsal process of the quadrate, and the high position of the subtympanic foramen close to the level of the dorsal margin of the skull roof. Although the squamosal of *Burkesuchus* is posteroventrally flexed as in baurusuchids, both the degree of flexure of the squamosal and its distal extent along the quadrate (i.e., almost to the level of the quadrate condyles) is more pronounced in the Chilean taxon. These features of the squamosal are absent among other crocodyliforms, thus they are here interpreted as autapomorphic for *Burkesuchus*. In the latter taxon, the external auditory meatus is only partially exposed in side view, different from the well-exposed condition of “protosuchians”, notosuchians and peirosaurids (e.g., *Araripesuchus, Hamadasuchus*^21^).

**Figure 7:**
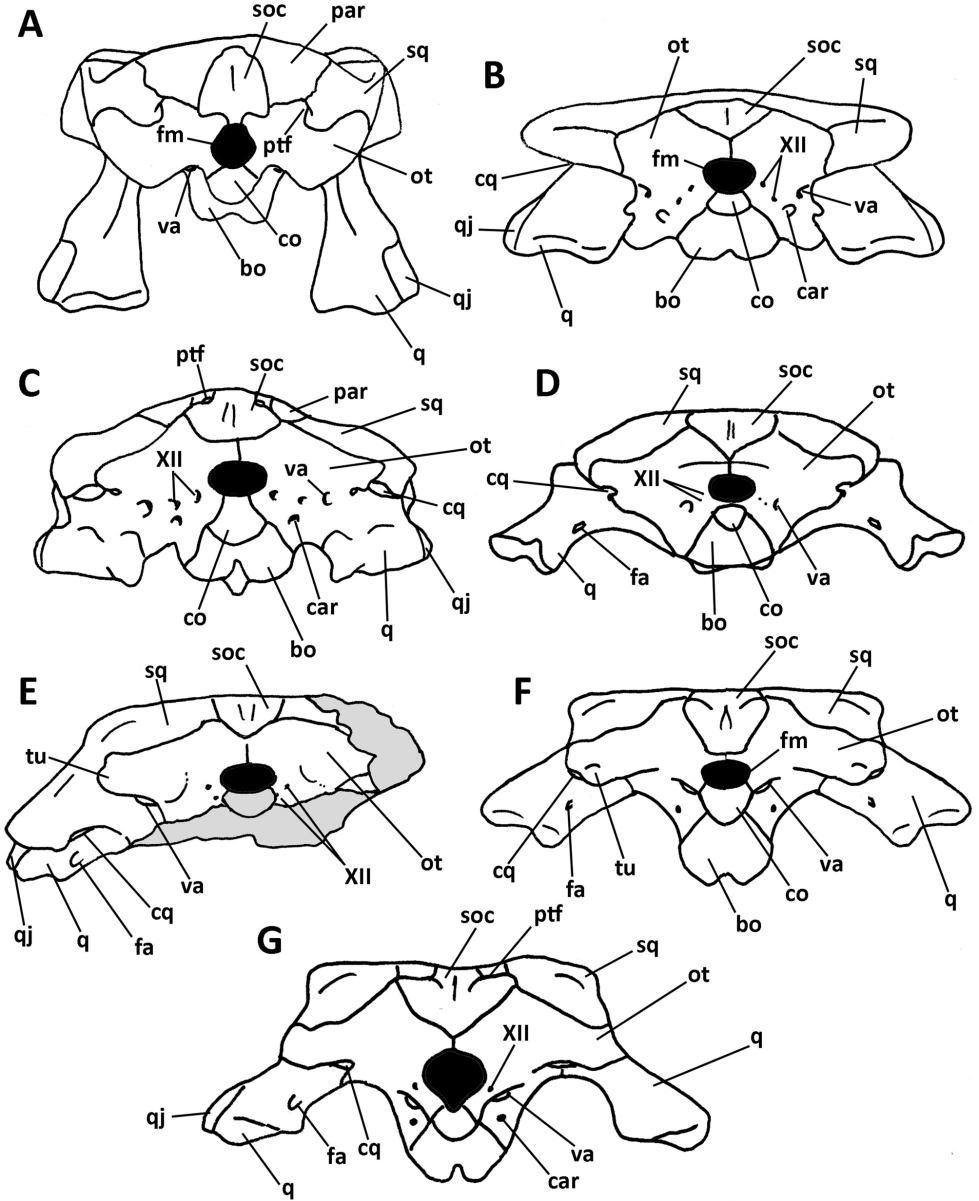
Occipital view of cranium of selected crocodyliforms. (**A**) *Sphenosuchus* (Sphenosuchia); (**B**) *Protosuchus* (“Protosuchia”); (**C**) *Pelagosaurus* (Thalattosuchia); (**D**) *Notosuchus* (Mesoeucrocodylia, Notosuchia); (**E**) *Burkesuchus* (Mesoeucrocodylia); (**F**) *Allodaposuchus* (Mesoeucrocodylia, Eusuchia); and (**G**) *Osteolaemus* (Mesoeucrocodylia, Neosuchia). bo, basioccipital; car, carotid foramen; co, occipital condyle; cq, cranioquadrate canal or foramen; fa, foramen aereum; fm, foramen magnum; ot, otoccipital; par, parietal; ptf, post-temporal fenestra; q, quadrate; qj, quadratojugal; soc, supraoccipital; sq, squamosal; tu, ventral tubercle of the paraoccipital process of otoccipital; va, vagus foramen; XII, foramen for cranial nerve XII. (**A**) modified from^57^; (**B**,**C**) modified from^34^; (**D**), modified from^58^; (**F**), modified and reconstructed from^27,39^; (**G**), modified from^40^. Not to scale.

*Burkesuchus* also differs from early neosuchians such as *Shamosuchus* and *Allodaposuchus*^38^ in that the latter exhibit a straight outer margin of squamosal. However, in these two neosuchians and *Burkesuchus* the squamosal bears a ventrally directed lamina that in crocodilians contacts the posterodorsal surface of the quadrate posteriorly, resulting in a bony enclosure of the meatal chamber^21^. In *Burkesuchus* as well as in some basal neosuchians (e.g., *Allodaposuchus, Goniopholis, Hylaeochampsa, Goniopholis, Anteophthalmosuchus*^3,39–41^) there exists a small otic aperture on the posterior surface of the cranium. In the above mentioned taxa both the squamosal and quadrate are not in contact posterior to the otic opening, thereby resulting in the formation of a cranioquadrate passage between the squamosal, quadrate, and exoccipital bones^21^ (Fig. 7). In extant crocodiles, the cranioquadrate passage is almost closed and represented by the cranioquadrate foramen that provides passage for one branch of cranial nerve VII, the orbitotemporal artery, and the lateral cephalic vein^42^.

Authors regard the closing of the cranioquadrate canal as diagnostic of mesoeucrocodylians^4,19^. However, in almost all eusuchians the cranioquadrate canal is delimited dorsally by the squamosal lamina, ventrally by the quadrate, and posteromedially by the otoccipital, whereas in neosuchians this canal is enclosed by the quadrate and otoccipital^19,39^. In *Burkesuchus* a small fissure separates the squamosal from the quadrate, leaving a slight, laterally opened cranioquadrate canal. Further, it has relatively small otoccipital and paraoccipital processes that are narrow and poorly laterally projected. In this way, the otoccipital does not form part of the cranioquadrate closure (Fig. 7). A similar condition was previously reported for goniopholidid neosuchians^39,41^. It is possible that the condition in *Burkesuchus* and goniopholidids represents an intermediate stage between the entirely opened meatal chamber of basal crocodyliforms (e.g., “protosuchians” and notosuchians) and the enclosed cranioquadrate foramen of extant crocodiles.

### *Burkesuchus* and its implications for the evolution of ear pneumaticity in crocodilians

The cranium of extant crocodiles is characterized by a pneumatic system that ontogenetically develops through the expansion of diverticula from the middle ear cavity. Diverticula penetrate most bones of the posterior part of the cranium and mandible, being linked with an elaborate system of cavities and tubes to the throat^42^. In “protosuchians” and notosuchians there are multiple subtympanic foramina that represent a plesiomorphic state for the clade^43^. It is known that in extant forms the single subtympanic foramen and its associated diverticulum have resonant functions^44^. Thus, the complex pneumatic morphology of basal crocodyliforms has been associated with an advanced auditory sensitivity and directionality^21,45^. In eusuchians the multiple foramina are reduced in the quadrate to just a single foramen^26^. This foramen represents the entrance of the siphonium, a hollow stem consisting of connective tissue and epithelium that contacts the articular bone of the mandible with the quadrate and continues through this bone until it exits through a foramen, the siphonial aperture, into the tympanic recess^46^. These extraordinary modifications are correlated with the acoustical coupling of both middle ears to aid in augmenting certain frequencies via a pressure difference, probably associated with the aquatic habits of living forms^45^. The presence of a single foramen on the quadrate for the entrance of the siphonium in *Burkesuchus* suggests that this complex siphonial system was already developed in this basal mesoeucrocodylian.

### *Burkesuchus* paleoecology

The anatomy of the braincase helps to recognize some palaeoecological features in *Burkesuchus* that may be important for understanding the habits of the first mesoeucrocodylians.

*Burkesuchus*, as in some other basal neosuchians such as *Allodaposuchus* and *Hylaeochampsa*, exhibits an enlarged posterior exposure of the squamosal and the retention of a ventral tubercle on the paraoccipital processes of the otoccipital, features that are indicative of a thick and robust anchoring of the *M. depressor mandibulae*, the only jaw abductor, and consequently a strong jaw opener^47,48^. However, the small size of the supratemporal fenestra and supratemporal foramen in *Burkesuchus* may indicate a more restricted attachment area for the adductor muscles of the jaw^4,49^. This kind of reduced supratemporal fenestra and fossae suggests a weaker force of jaw adduction for catching, killing and tearing large prey^47,50^. In living crocodylians, the capability of catching, killing and tearing large prey is accompanied by the capacity for torsional feeding and a stronger bite for holding and crushing prey during rolling, which necessarily includes an increasing need for more powerful adductor musculature^4^. It is usually regarded that strong musculature associated with holding prey by rolling is correlated with a solid rostrum and extensive secondary palate in eusuchians^4,49^. *Burkesuchus*, in having relatively small supratemporal fossae and supratemporal fenestra, suggests that rostrum and palate adaptations typical of eusuchian crocodiles were probably absent in this basal mesoeucrocodylian.

The postcranial anatomy of *Burkesuchus* provides some inferences on its ecology. As noted in the description, the scapulocoracoid glenoid shows an intermediate condition between basal crocodyliforms and modern crocodilians. However, the presence of a prominent lip on the scapular facet and the posteriorly oriented coracoid facet suggest that the humerus was unable of important dorsal excursion and points to a plesiomorphically more upright posture of the forelimb when compared with extant crocodilians^6,51^.

In contrast with the forelimb, the femur of *Burkesuchus* closely resembles the condition of extant crocodiles. This element shows an accentuated sigmoid curvature (contrasting with the straighter condition of more basal crocodilians and notosuchians) and strongly asymmetrical distal end, features correlated with a sprawling posture^52,53^.

In this sense, the presence of a well-developed fourth trochanter, basitrochanteric fossa and muscle scars indicate well-developed *Mm. caudofemoralis longus* and *brevis* (see^52,54^). This contrasts with the condition of basal crocodyliforms and notosuchians in which these scars are not prominent^31^. Its reduction is usually correlated with progressive reduction of the tail-based musculature and reflects a knee-based limb retraction of upright posture and parasagittal gait^56–57^. In modern crocodiles the caudofemoral musculature is a critical component of the locomotor apparatus, because it produces wide arcs of femoral retraction and is important on femoral rotation^55^.

The femur of *Burkesuchus* shows features indicative of a sprawling stance and gait used by modern crocodiles when swimming and upon entering the water, as well as tail-based locomotion of extant crocodilians in water^32^. These features and behaviors were probably already present in *Burkesuchus*. By contrast, the forelimb indicates a more upright posture than shown in extant crocodiles. It is possible that the acquisition of sprawling posture in crocodilians was not acquired simultaneously in both fore- and hind limbs, but was decoupled. Although speculative, it is possible that the hind limbs, which tend to be more important for locomotion in living crocodiles, changed their shape before the forelimbs. It is possible that *Burkesuchus* illustrates this intermediate condition with somewhat upright forelimbs and sprawling hind limbs.

In sum, phylogenetic analysis supports *Burkesuchus* as a basal member of Mesoeucrocodylia, thus expanding the meagre record of non-pelagic representatives of this clade for the Jurassic Period. Previously recorded members of non-pelagic Jurassic Mesoeucrocodylia are the presumably fresh-water Atoposauridae, Goniopholididae and Paralligatoridae (i.e., *Batrachomimus*).

Interestingly, although *Burkesuchus* is depicted phylogenetically closer to neosuchians than notosuchians and baurusuchians, it exhibits several plesiomorphic features resembling “protosuchians” (e.g., cranium table transversely wide, acromial process prominent, deltopectoral crest anteromedially projected), in conjunction with highly derived features (e.g., inflected squamosal posteriorly closing the meatal chamber, femur distally asymmetrical, osteoderms proportionally large). The autapomorphic condition of the posteroventral wing of squamosal and the excavation of the anterodorsal end of quadratojugal suggests the presence of well-developed earlids.

*Burkesuchus* expands the taxonomic diversity of Jurassic crocodylomorphs. Nevertheless, its body size falls within the size range (i.e., less than 1 m in whole length) that was usual for most Triassic and Jurassic terrestrial crocodyliforms. The position of *Burkesuchus* among mesoeucrocodylians, in tandem with other basal members of this clade, such as the aquatic Atoposauridae, indicates that basal neosuchians also diversified in the context of small body size regime. This constraint on body size was released in marine forms of Jurassic age, as well as in different clades during the Cretaceous (the terrestrial baurusuchids, peirosaurids and sphagesaurids, and the fresh-water pholidosaurids^58^). We are unable to explain the biological reasons for the retention of small sizes among basal terrestrial crocodyliforms, but competition with ecologically dominant theropod dinosaurs cannot be ruled out.

*Burkesuchus* constitutes one of the few records of non-aquatic, mesoeucrocodylians for the Jurassic Period, and alongside the advanced neosuchian *Batrachomimus*, from Pastos Bons Formation, NE Brazil^10^, supports the idea that South America is crucial for evaluating further the radiation and evolution of crocodyliforms during the Late Jurassic.

## Supplementary Information

Supplementary Information 1.

Supplementary Information 2.
